# The Feasibility of Targeted Magnetic Iron Oxide Nanoagent for Noninvasive IgA Nephropathy Diagnosis

**DOI:** 10.3389/fbioe.2021.755692

**Published:** 2021-11-25

**Authors:** Yaoyao Wu, Qiang Huang, Junli Wang, Yuhua Dai, Ming Xiao, Yangyang Li, Hongbo Zhang, Wenbo Xiao

**Affiliations:** ^1^ Department of Radiology, First Affiliated Hospital, School of Medicine, Zhejiang University, Hangzhou, China; ^2^ Department of Radiology, Fourth Affiliated Hospital, School of Medicine, Zhejiang University, Hangzhou, China; ^3^ Clinical Medical Research Center, Fourth Affiliated Hospital, School of Medicine, Zhejiang University, Hangzhou, China; ^4^ Department of Pathology, First Affiliated Hospital, School of Medicine, Zhejiang University, Hangzhou, China; ^5^ Zhejiang Provincial Key Laboratory for Precision Diagnosis and Treatment of Major Gynecological Diseases, Women’s Hospital, Zhejiang University School of Medicine, Hangzhou, China; ^6^ Pharmaceutical Sciences Laboratory, Åbo Akademi University, Turku Bioscience Centre, University of Turku, Åbo Akademi University, Turku, Finland

**Keywords:** IgA nephropathy (IgAN), Fe3O4-RGD, αvβ3-targeted, noninvasively diagnosis, T2 weighted MR imaging

## Abstract

IgA nephropathy is the most common glomerular disease in the world and has become a serious threat to human health. Accurate and non-invasive molecular imaging to detect and recognize the IgA nephropathy is critical for the subsequent timely treatment; otherwise, it may progress to end-stage renal disease and lead to glomerular dysfunction. In this study, we have developed a sensitive, specific, and biocompatible integrin αvβ3-targeted superparamagnetic Fe_3_O_4_ nanoparticles (NPs) for the noninvasive magnetic resonance imaging (MRI) of integrin αvβ3, which is overexpressed in glomerular mesangial region of IgA nephropathy. The rat model of IgA nephropathy was successfully established and verified by biochemical tests and histological staining. Meanwhile, the clinical ^18^F-AlF-NOTA-PRGD2 probe molecule was utilized to visualize and further confirmed the IgA nephropathy *in vivo via* positron emission computed tomography. Subsequently, the Fe_3_O_4_ NPs were conjugated with arginine–glycine–aspartic acid (RGD) molecules (Fe_3_O_4_-RGD), and their integrin αvβ3-targeted T2-weighted imaging (T2WI) potential has been carefully evaluated. The Fe_3_O_4_-RGD demonstrated great relaxation *in vivo*. The T2WI signal of renal layers in the targeted group at 3 h after intravenous injection of Fe_3_O_4_-RGD was distinctly lower than baseline, indicating MRI signal decreased in the established IgA nephropathy rat model. Moreover, the TEM characterization and Prussian blue staining confirmed that the Fe_3_O_4_-RGD was located at the region of glomerulus and tubular interstitium. Moreover, no obvious signal decreased was detected in the untargeted Fe_3_O_4_ treated and normal groups. Collectively, our results establish the possibility of Fe_3_O_4_-RGD serving as a feasible MRI agent for the noninvasive diagnosis of IgA nephropathy.

## Introduction

IgA nephropathy is the most common glomerular disease in the world, and approximately 50% of IgA nephropathy will progress to end-stage renal disease within 30 years regardless of treatment (Moriyama et al., 2014). The gold standard for the diagnosis of IgA nephropathy is renal biopsy, clinically (Caliskan and Kiryluk, 2014). However, renal biopsy suffers from many disadvantages, for example, as an invasive examination, renal biopsy may cause various complications, such as perirenal hematoma and pain (Marek-Bukowiec et al., 2018). Moreover, biopsy can only reflect the short-term status of the disease, and multiple biopsies cannot be carried out generally. Hence, the dynamic pathological changes of the disease cannot be evaluated at real time (Suzuki, 2019). Besides, biopsy may not be performed normally due to physical factors, such as hypertension, anatomical variation, and pregnancy. Thus, non-invasive detection of IgA nephropathy has become an urgent issue. Previous studies have tried to find biomarkers of IgA nephropathy from serum and urine samples, such as increasing galactose-deficient IgA1 (Gd-IgA1) in serum (Caliskan and Kiryluk, 2014), decreasing mRNA level of IFI27 protein in peripheral blood mononuclear cells (Nagasawa et al., 2016), and combination of seven urinary markers (Neprasova et al., 2016). However, those markers are not widely used in clinic due to the lack of more experiments to validate effectiveness. Therefore, non-invasive and dynamic diagnostic approaches of IgA nephropathy is still urgent.

Integrins are heterodimer transmembrane glycoprotein receptors composed of two subunits a and *β* (Jin et al., 1996; Pozzi and Zent, 2013), which can activate intracellular and extracellular signal pathways by combining with extracellular matrix such as fibronectin, collagen, laminin, or receptors on other cell surface, and participate in cellular proliferation, differentiation, adhesion, as well as migration (Takada et al., 2007; Anthis and Campbell, 2011). Integrin αvβ3, as a vitronectin receptor, is rarely expressed in normal tissues, and a little expression can be observed around glomerular capillary loops, mesangial regions, and podocytes (Jin et al., 1996; Gauer et al., 1997; Amann et al., 2012). Several studies demonstrated the high expression of integrin αvβ3 in IgA nephropathy, which can be mainly observed in the expanded mesangial region (Peruzzi et al., 2000; Du et al., 2012). It well known that arginine–glycine–aspartic acid (RGD) is a small molecular peptide and has a high affinity for integrin αvβ3 (Ruoslahti, 1996). Targeted RGD peptides can be used to estimate the expression of integrin αvβ3 quantitatively or semi-quantitatively, which has been widely used in cancer research about evaluating angiogenesis, early detection, and assessment of therapeutic response (Lang et al., 2011; Chen et al., 2020).

Magnetic resonance imaging (MRI), as a common examination for clinical disease diagnosis, possesses high spatial resolution and good tissue contrast and is not radioactive (Ye et al., 2002; Jiang et al., 2009). Nevertheless, the shortcoming of MRI is relative low sensitivity (Jiang et al., 2009). Superparamagnetic iron oxide (SPIO), as a commonly used T2 contrast agent, is able to make a rapid response to an external magnetic field, and can significantly reduce the tissue signal intensity in T2-weighted imaging and achieve more signal changes, which can overcome low sensitivity of MRI at some degree (Ye et al., 2002; Sargsyan et al., 2012; Wen et al., 2014). Furthermore, SPIO conjugating with special molecules can target specific receptors *in vivo* and demonstrated great biocompatibility (Sargsyan et al., 2012). At present, a series of studies have investigated the feasibility of targeting molecules to detect renal diseases, such as anti-C59-b-SPIO was used for the detection of Heymann nephritis (Huang et al., 2015) and CR2-SPIO was designed for the detection of lupus nephritis (Serkova et al., 2010; Sargsyan et al., 2012). However, there are few studies to investigate the feasibility of SPIO-RGD nanoprobe for detecting and monitoring IgA nephropathy in a non-invasive way.

Meanwhile, as an important molecular imaging technique, positron emission computed tomography (PET) possesses superior sensitivity and can be used to observe the distribution of specific tracers as well as quantitatively measure the transport rates *in vivo* (Glaser et al., 2008; Guo et al., 2012a) ^18^F is a popular PET radioisotope owing to its short half-life (109.8 min) and low positron energy (0.64 MeV) (Cheng et al., 2015). The application of ^18^F in the detection of renal disease involves not only neoplastic lesions, but also non-neoplastic lesions. Patients with drug-related acute renal interstitial nephritis showed higher ^18^F-FDG uptake in the renal cortex than baseline (Katagiri et al., 2010; Qualls et al., 2019). Moreover, ^18^F-labeled RGD tracers can be utilized to target integrin αvβ3 for specific targeting diagnosis (Cheng et al., 2015; Zhang et al., 2016; Jin et al., 2017; Li et al., 2019). Particularly, ^18^F-AlF-NOTA-PRGD2 has become an important PET tracer to measure the expression of integrin αvβ3 (Gao et al., 2012; Guo et al., 2012b). However, the short physical half-life of ^18^F PET probe requires experiment in a short time; otherwise, the image quality may be poor due to the rapid attenuation of ^18^F. In addition, the ionizing radiation of ^18^F may cause the body damage in the process used. Besides, an inherent limitation of PET imaging is that the spatial resolution is lower compared with MRI. The renal tissue such as cortex and medulla cannot be revealed distinctly. Nevertheless, in this work, ^18^F-AlF-NOTA-PRGD2 probe may be used to auxiliary confirm the successful establishment of the mouse model of IgA nephropathy.

**SCHEME 1:**
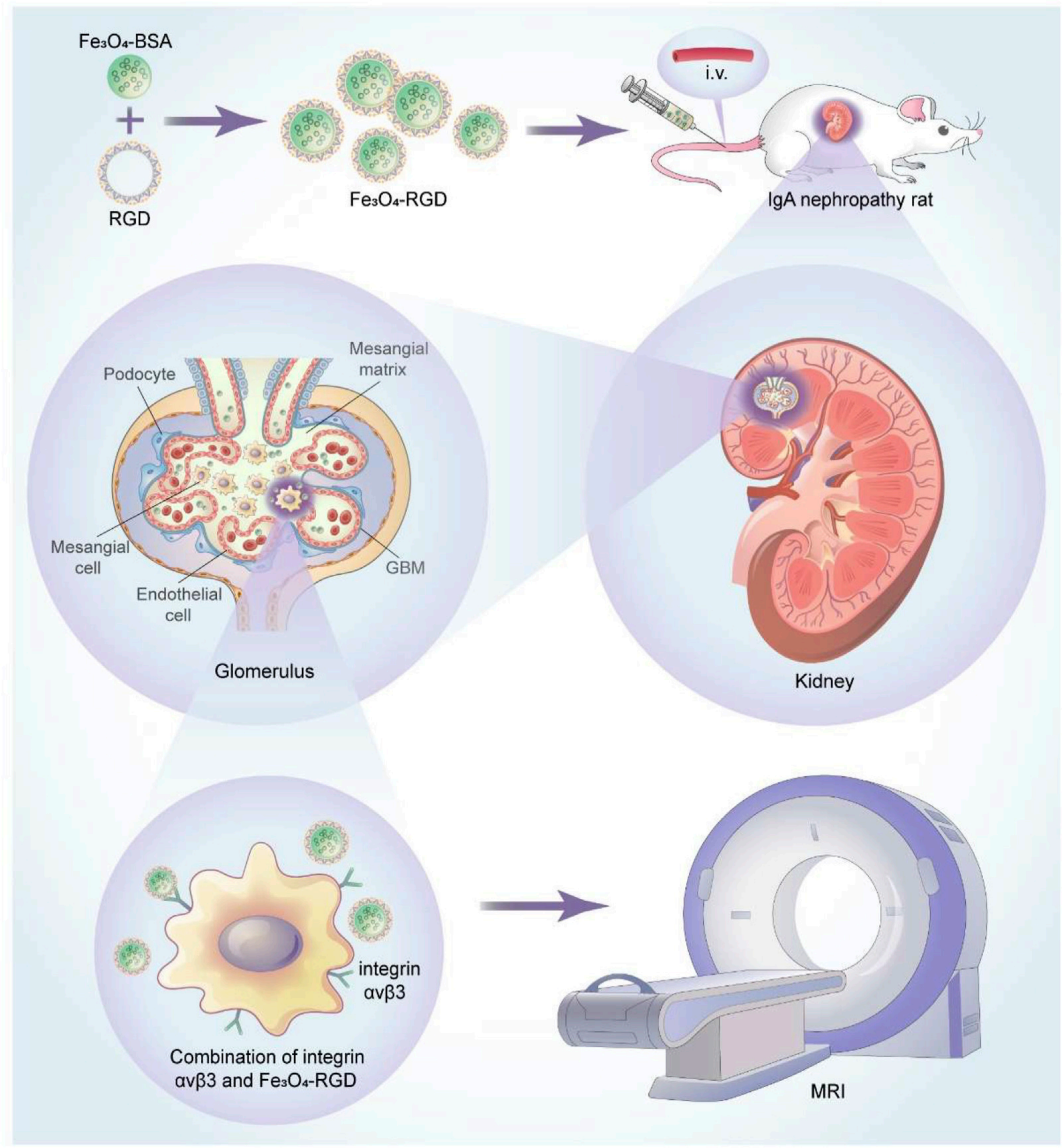
Schematic illustration of the application of Fe_3_O_4_-RGD NPs for diagnosis of IgA nephropathy.

In our work, a facile method was used to prepare the uniform BSA-modified Fe_3_O_4_ nanoparticles (NPs). Subsequently, these paramagnetic NPs were functionalized with RGD molecules (RGD-Fe_3_O_4_) as an integrin αvβ3-targeting MRI nanoprobe to specifically recognize the glomerular mesangial region of IgA nephropathy (Scheme 1). At first, the rat model of IgA nephropathy was successfully established and verified by biochemical tests and histological staining. Furthermore, the clinical ^18^F-AlF-NOTA-PRGD2 probe molecule was utilized to confirm the successful establishment of the IgA nephropathy rat model. Then, the synthesized RGD-Fe_3_O_4_ NPs were injected intravenously into rats and the integrin αvβ3-targeted T2-weighted imaging (T2WI) RGD-Fe_3_O_4_ NPs have been carefully evaluated in the IgA nephropathy rat model. Therefore, our study provided a foundation into the development of safe and effective T2WI for molecular imaging of detecting IgA nephropathy noninvasively.

## Materials and Methods

### Synthesis and RGD Surface Modification

Fe_3_O_4_ NPs were firstly synthesized by a simple wet chemistry method. Briefly, 200 ml of 0.18 mol/L Fe (NO_3_)_3_ solution and 200 ml of 0.12 mol/L FeSO_4_ were mixed homogeneously. The above mixed solution was kept stirred at 40^o^C under the protection of N_2_ atmosphere. Subsequently, 48 ml of NH_3_.H_2_O solution was added and stirred for 15 min and then aged at 40^o^C for 5 min. Finally, Fe_3_O_4_ magnetic NPs were obtained by centrifugation and washing process. Fe_3_O_4_ magnetic NPs were then modified with BSA molecules. Specifically, an appropriate amount of Fe_3_O_4_ NP solution was added to 30 ml of BSA (10 mg/ml) solution and stirred for 24 h, mechanically. The Fe_3_O_4_-BSA NPs were obtained by separating and washing processes.

Twenty milliliters of Fe_3_O_4_-BSA NP solution (2 mg/ml) was uniformly dispersed in 28 ml of ultra-pure water, and then followed by adding 2 ml of PBS (pH 7.4) solution. The above solution was subjected to further ultrasonic dispersion for 10 min. Subsequently, 230 mg of EDC was added to the above mixed solution and stirred for 0.5 h in the dark. One hundred fifty milligrams of NHS was further added and vigorously stirred for 1 h under dark conditions. Fifteen milliliters of RGD (1 mg/ml) was then added and stirred for 24 h under dark conditions, continually. Finally, the above solution was centrifuged through an ultrafiltration tube at a speed of 5,500 RPM and washed three times with ultra-pure water. Fe_3_O_4_-BSA-RGD NPs were further dispersed in 20 ml of ultra-pure water for further use.

### Establishment of Animal Models

For animal model construction about IgA nephropathy, 40 male SD rats (6 to 8 weeks old) were purchased from Zhejiang Academy of Medical Sciences and fed at 22.8^o^C (room temperature) and 59.6% relative humidity in the SPF degree animal laboratory of the First Affiliated Hospital, School of Medicine, Zhejiang University. All rats were divided into two groups randomly (model group and control group). The rats of the model group were administrated with 100 g/L BSA solution every other day in a dose of 800 mg/kg by gavage. Besides, the model group also received 0.4 ml mixed solution of CCl4 and castor oil weekly by subcutaneous injection, and 0.05 mg of LPS every other week by tail vein injection. Simultaneously, isodose saline was administered to the control group in the same way.

After 6 weeks’ feeding, biochemical and pathological examinations were conducted to verify histopathological changes of IgA nephropathy and investigate renal functions in the above two groups. The samples were harvested repeatedly every 2 weeks until the model group got a significant difference in histology compared with the control group.

### Biochemical Tests and Histological Staining

To detect renal functions, blood samples were taken from the tail vein and 24-h urine was collected to determine creatinine (CRE), blood urea nitrogen (BUN), and albumin (ALB) levels by automatic biochemical analyzers (LW C400, LANDWIND, Shenzhen, China and Chemray240, Rayto, Shenzhen, China).

To compare the changes of mesangial cells and matrix in two groups, two rats from the model and control group were sacrificed, and one part of sagittal renal tissue slices was fixed in formalin, embedded in paraffin, sliced, and dewaxed to water for HE, PAS, and MASSON staining. Subsequently, staining images were acquired from the light microscope (Eclipse Ci, NIKON, Tokyo, Japan).

### Micro-PET Imaging *In Vivo*

In order to further confirm successful establishment of the IgA nephropathy rat model, the ^18^F-AlF-NOTA-PRGD2 probe molecule for micro-PET technology was used. The difference in renal radioactivity uptake between IgA nephropathy rat and the normal group was compared after intravenous application of ^18^F-ALF-NOTA-PRGD2 *in vivo*. Rats (*n* = 1/group) were weighed and anesthetized with 4% chloral hydrate by intraperitoneal injection. Micro-PET was performed at 30, 40, 50, 60, 70, 80, and 90 min after injection of ^18^F-AlF-NOTA-PRGD2 (about 37 mBq) *via* tail vein. The images were reconstructed according to a two-dimensional Ordered Subsets Expectation Maximum (OSEM) algorithm and then were processed by the Inveon Research Workspace (IRW). The regions of interest (ROIs) of the entire renal parenchyma were drawn manually, and maximum percent injected dose per gram of body weight (%ID/g) was obtained on the workstation directly.

### Fluorescence Staining

To confirm the increased expression of integrin avβ3 in model rat kidney, integrin avβ3 fluorescence staining was performed. Firstly, renal tissue was fixed, embedded, sliced, and dewaxed to water conventionally, and then soaked into 3% H_2_O_2_ solution for 10–20 min. Subsequently, renal sections were treated with citric acid buffer for 15–20 min and sealed with 10% normal serum for 30 min at 37^o^C. After that, the diluted primary antibody (anti-Integrin alpha V beta three antibody, ab7166) was added and incubated at 37^o^C for 60 min. The fluorescent second antibody was added and incubated at 37^o^C for 30 min. DAPI was added for core staining and section sealing. In the end, the fluorescence images were shot under a fluorescence microscope (Eclipse Ti, NIKON, Tokyo, Japan). It is worth mentioning that quantitative analysis of immunofluorescence intensity was performed by Image-Pro Plus 6.0 (Media Cybernetics, Inc. Rockville, United States). Briefly, the mean density, which was the ratio of integrated option density to area, was acquired to estimate the fluorescence intensity.

### MRI Evaluation *In Vitro* and *In Vivo*

For the purpose of evaluating relativity r2 of Fe_3_O_4_-RGD *in vitro*, a series of Fe_3_O_4_-RGD solutions with different Fe concentrations (0, 0.034, 0.067, 0.135, 0.270, 0.540, 1.080) were prepared. Then, T2-weighted imaging and T2*mapping were performed on the clinical GE 3.0-T MRI (DiscoveryMR750, GE Medical System, Boston, United States). Subsequently, the value of R2 was measured by READ Y View (AW VolumeShore7, GE Medical System, Boston, United States) based on the T2* mapping. Finally, the relative curve between R2 value and Fe_3_O_4_-RGD gradient concentration was fitted by GraphPad Prism 8 (GraphPad Software Inc., San Diego, CA, United States).

In order to explore the feasibility for the early noninvasive diagnosis of IgA nephropathy through identifying the increased expression of integrin avβ3 early, the relative rat MRI experiment was performed. The rats were divided into three groups: Group 1 (targeted group) received intravenous tail injection of Fe_3_O_4_-RGD solution at a dose of 15 mg Fe/kg. Group 2 (untargeted group) received intravenous tail injection of Fe_3_O_4_-BSA solution at a dose of 15 mg Fe/kg. Group 3 (normal) group received intravenous tail injection of Fe_3_O_4_-RGD solution at a dose of 15 mg Fe/kg.

Subsequently, the above three group’s rats were weighted and anesthetized with 4% chloral hydrate at a dose of 0.8 ml/kg; T2-weighted imaging and T2* mapping were performed at baseline and 3 h after administering Fe_3_O_4_-RGD or Fe_3_O_4_-BSA solution through the tail vein on the clinical GE 3.0-T MRI (DiscoveryMR750, GE Medical System, Boston, United States). T2-weighted imaging was acquired with the following parameters: repetition time (TR), 3,000 ms; echo time (TE), 68 ms; flip angle, 142°; field of view (FOV), 8 cm × 8 cm; matrix size, 128 × 128; number of sections, 16; slice thickness, 1.0 mm; total acquisition time, 4 min. The parameters of T2* mapping were as follows: repetition time (TR), 110 ms; echo time (TE), 2.0 to 16.1 ms; flip angle, 20°; field of view (FOV), 9 cm × 9 cm; matrix size, 64 × 64; number of sections, eight; slice thickness, 2.2 mm; total acquisition time, 30 s; the R2 values of renal different layers between groups were measured and statistical analysis were conducted by SPSS 26 (IBM Corp, Armonk, NY).

After MRI scan, those rats were sacrificed through cervical dislocation. Kidney samples were harvested rapidly and fixed in formalin for Prussian blue staining, as well fixed in 2.5% glutaraldehyde for TEM tests, respectively.

## Results and Discussion

### Synthesis and Characterization of Fe_3_O_4_-BSA NPs

**FIGURE 1:**
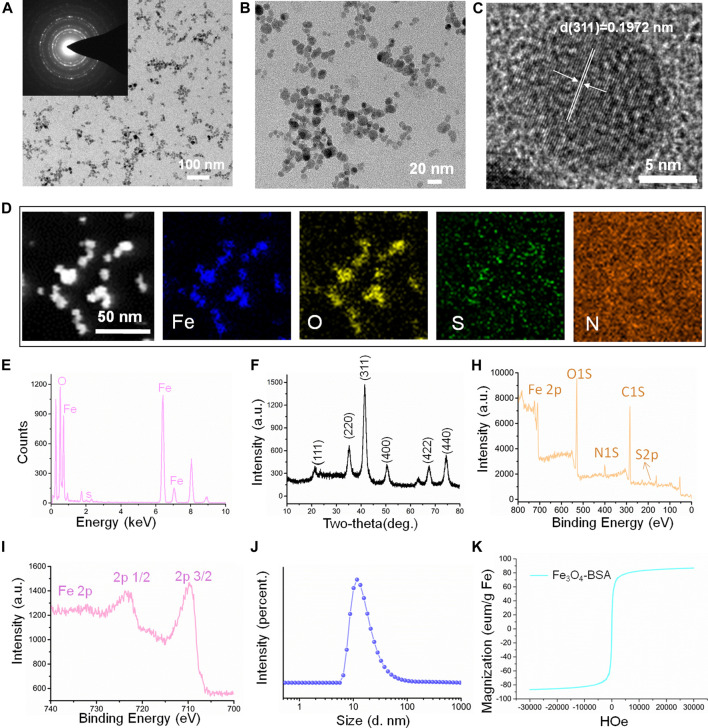
Characterization of the magnetic nanoparticles (Fe_3_O_4_-BSA NPs). **(A, B)** TEM and magnified TEM images of Fe_3_O_4_-BSA NPs (the inset image is the corresponding SAED pattern). **(C)** HRTEM image. **(D, E)** HAADF-STEM elemental mapping images and EDX patterns of Fe_3_O_4_-BSA NPs. **(F)** X-ray diffraction pattern. **(H, I)** XPS spectrum of Fe_3_O_4_-BSA NPs and spectra of Fe2p orbits for the binding energy curve. **(J)** Size distribution of Fe_3_O_4_-BSA NPs in ultrapure aqueous solution. **(K)** The MH hysteresis curve of Fe_3_O_4_-BSA NPs.

The Fe_3_O_4_ magnetic NPs were initially prepared and then modified with BSA molecule (Fe_3_O_4_-BSA) for further RGD grafting process. The morphology and structure were investigated by transmission electron microscopy (TEM). Figures 1A, B demonstrated that the Fe_3_O_4_-BSA NPs exhibited a regularly spherical morphology with the uniform diameters in the range ∼20 nm. Meanwhile, the insert selected area electron diffraction (SAED) pattern demonstrated the defined diffraction rings, implying the polycrystalline nature of Fe_3_O_4_-BSA. Furthermore, the HRTEM images of Fe_3_O_4_-BSA NPs revealed that the distance between the adjacent lattice fringes is ∼0.1972 nm (Figure 1C), which agrees well with the crystal face of (311). The elemental mappings indicated that Fe, O, S and N elements distributed homogeneously within the Fe_3_O_4_-BSA matrix (Figure 1D). All expected essential chemical elements (Fe, O, and S) were verified by the energy dispersive x-ray (EDX) spectrum (Figure 1E). The crystalline nature Fe_3_O_4_-BSA NPs were further determined by x-ray diffraction (XRD). As presented in Figure 1F **,** the characteristic peaks of Fe_3_O_4_-BSA are related to the crystal planes of Fe_3_O_4_ crystal (JCPDS Card No. 19–0629) (Atila Dincer et al., 2019), indicating that the crystal structures of Fe_3_O_4_ remained unchanged after modification. Subsequently, the chemical composition and the surface states of Fe_3_O_4_-BSA were investigated by x-ray photoelectron spectroscopy (XPS). As displayed, the XPS spectrum of Fe_3_O_4_-BSA contains C1s, O1s, N 1s, S 2p and Fe 2p peaks (Figure 1H). In the Fe 2p spectrum (Figure 1I), the main peaks are at approximately 710.4 and 723.6 eV (Lian et al., 2019). One notable fact is that the hydrodynamic dimensions of Fe_3_O_4_-BSA is ∼20 nm and the NP solution possessed narrow size distribution and good dispersion (Figure 1J), indicating the good dispersity and uniform size of the Fe_3_O_4_-BSA NPs. The room-temperature hysteresis loop of the Fe_3_O_4_-BSA NPs was further measured. The magnetization curves of the samples are exhibited in Figure 1K; the Fe_3_O_4_-BSA NPs demonstrated remarkable superparamagnetic properties and saturation magnetization was 86.8 emu/g. To investigate the targeting performance of Fe_3_O_4_-BSA NPs for IgA nephropathy detection, RGD peptide, a targetable molecule for integrin ανβ3 molecule, was modified onto Fe_3_O_4_-BSA NPs (Fe_3_O_4_-RGD), which were determined by TEM and EDS mapping observed results. As demonstrated in Supplementary Figure S1, the Fe_3_O_4_-RGD NPs possess a well-defined spherical morphology with a diameter of approximately 20 nm and contains Fe, O, S, and N elements. A more interesting phenomenon is that the N element signal intensity was enhanced compared with the Fe_3_O_4_-BSA, which may be due to the successful grafting of the RGD molecules. The hydrodynamic dimensions of Fe_3_O_4_-RGD NPs increased from ∼20 to ∼50 nm (Supplementary Figure S2A). Meanwhile, the zeta potential changed from ∼−30.3 to ∼22.9 mV after the RGD modification due to the mildly positive charged RGD molecule (Supplementary Figure S2B, C). Moreover, the Fe_3_O_4_-RGD NPs can be well dispersed in pure water, PBS, FBS, and DMEM solution (Figure S3). Furthermore, the cytotoxicity of Fe_3_O_4_-RGD on normal cells (293T cells) was assessed by CCK-8 test *in vitro*. The result showed that the cells’ viability slightly decreased with the increase in concentrations after 24-h incubation. All groups demonstrated low cytotoxicity compared with the control group (*p* > 0.05). About 74% cell viability was still observed at the highest concentration (80 μg/ml) of Fe_3_O_4_-RGD (Supplementary Figure S4). This result suggested that Fe_3_O_4_-RGD NPs possess relative biocompatibility. Finally, the hemolysis assay exhibited obvious red color of positive control compared with the faint-yellow liquid of Fe_3_O_4_-RGD sample groups and negative control (Supplementary Figure S5), which indicated that the hemolysis of positive control was more significant than Fe_3_O_4_-RGD groups and negative control. The quantitative results of absorbance confirmed the result again. The mean values of hemolysis ratio for all Fe_3_O_4_-RGD samples were lower than 5%, which reached the standard of national biological safety for medical materials. These results suggested the blood biocompatibility of Fe_3_O_4_-RGD and is feasible to conduct animal experiments.

### Establishment of the Animal Model

#### Biochemical Tests

**FIGURE 2:**
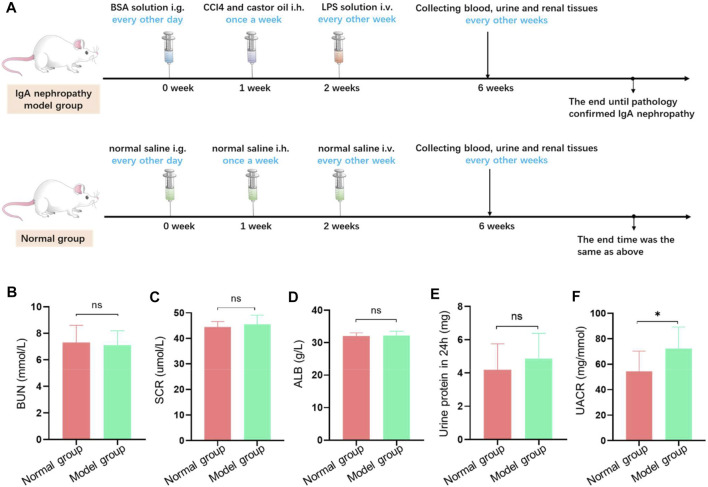
Th**e** establishment of IgA nephropathy rat. **(A)** The scheme of dosing process to establish IgA nephropathy rat. **(B–F)** Biochemical test examination of rats with different treatment groups. There was no significant difference in blood bun **(B)**, blood Cr **(C)**, blood ALB **(D)**, and total 24-h urinary protein **(E)** between the model group and the normal group. Meanwhile, there was significant difference in the ratio of urinary albumin to creatinine concentration **(F)** between the model group and the normal group (*p < 0.05).

To compare the discrepancy in renal function between the model group and control group, a series of renal functional indexes were detected. The detailed experimental process is presented in Figure 2A. The results demonstrated that there were no significant differences in serum creatinine (Cr), blood urea nitrogen (BUN), serum albumin (Alb), and 24-h urinary protein (*p* > 0.05) among two groups (Figures 2B–E). In contrast, the ratio of urinary albumin and creatinine was higher in the model group compared with the control group (*p* < 0.05) (Figure 2F). This result suggested that the pathological changes of model rats was in the primary stage and showed mild impairment of renal function.

### Histological Tests

**FIGURE 3:**
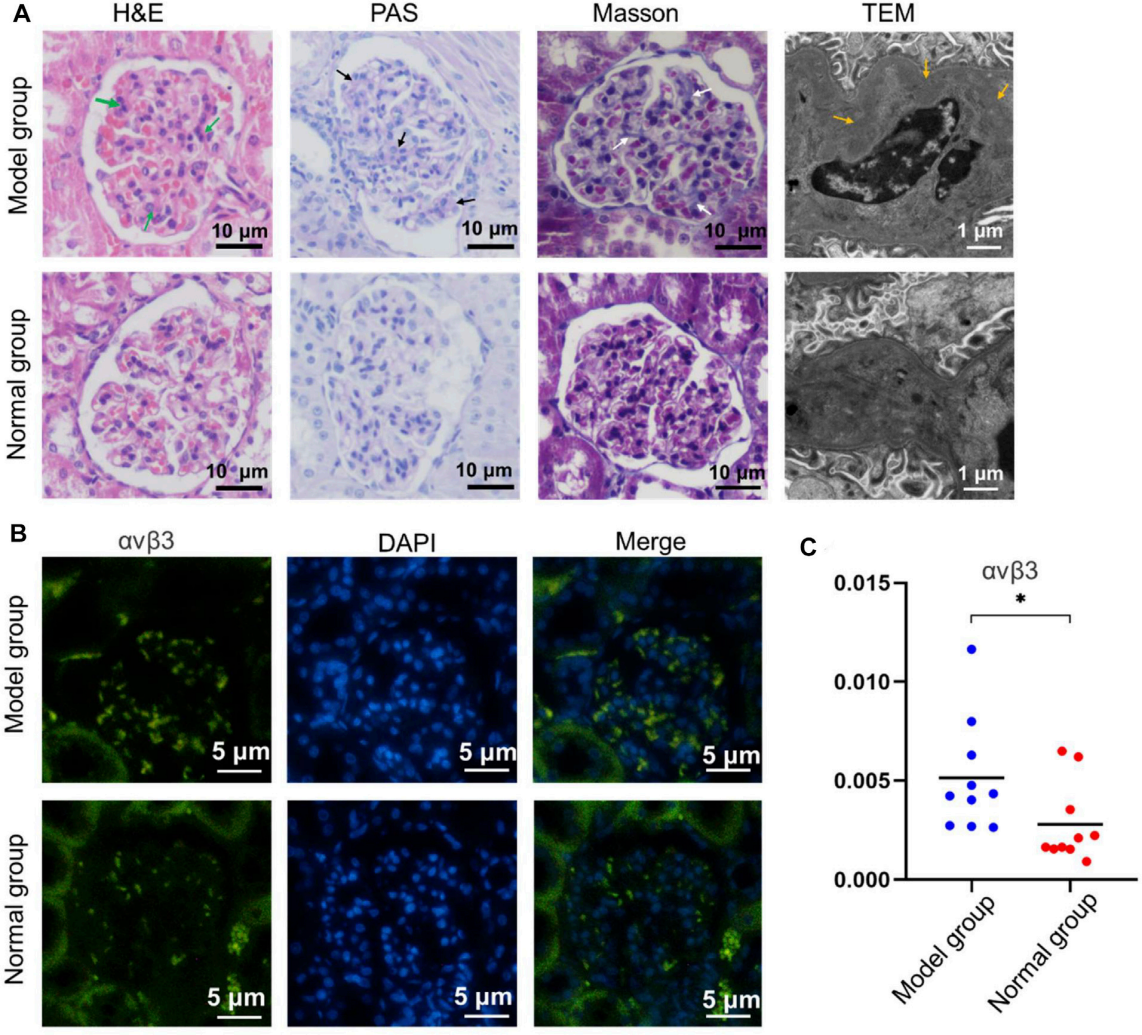
Pathological staining of renal tissue in the model group and normal group. **(A)** The H&E staining, PAS staining, Masson staining, and TEM investigation were studied, respectively. **(B)** αvβ3 Immunofluorescence of renal tissue with different treatment. **(C)** The corresponding quantitative analysis of the expressed αvβ3 in (b) (*p < 0.05).

To determine the histopathological changes of IgA nephropathy in the model group, various histological tests were conducted. H&E staining revealed that lots of mesangial cells were observed among expanded mesangial matrix with slightly compressed capillary loop in the model group, which was not obvious in the normal group (Figure 3A). Meanwhile, compared with the control group, the red and blue stained region means the mesangial area was widened and deepened in the model group on PAS staining (Figure 3A) and Masson staining (Figure 3A), respectively. There were electron-dense deposits (yellow arrow) in the glomerular mesangial area in the model group under TEM image, while no electron-dense deposits were seen in the normal group. These results indicated that model rats presented an increase in glomerular mesangial cells, mesangial matrix, and immune deposits, corresponding to the pathological changes of IgA nephropathy depicted in the literature (Caliskan and Kiryluk, 2014), and preliminarily demonstrated that the animal model was successfully constructed.

### Immunofluorescence Staining

Relevant immunofluorescence staining experiment was performed to investigate the expression of integrin αvβ3 in renal tissues between the model and normal group. The results illustrated that there was a large amount of spotted green fluorescence presented in the glomerular mesangial area in the model group, and only little green fluorescence in the mesangial region in the normal group (Figure 3B). The semi-quantitative analysis of fluorescence intensity demonstrated that the mean optical density in the model group was higher than that of the normal group (*p* < 0.05) (Figure 3C). This indicated that the expression of integrin αvβ3 in model rat kidney was higher than that in the normal group. Moreover, the IgA immunofluorescence staining of renal tissue in the model group or normal group was also investigated. There was a large amount of IgA immune complex deposition in the glomerular mesangial area of the model group. Contrarily, a small amount of IgA immune complex deposition in the glomerular mesangial area of the normal group was found (Supplementary Figure S6). Therefore, the above immunofluorescence staining results could be the basis for the later study of targeting renal integrin αvβ3 by special contrast agent to achieve imaging diagnosis of IgA nephropathy disease.

### Micro-PET Imaging

In this study, the ^18^F-AlF-NOTA-PRGD2 probe was then used to confirm the successful establishment of the IgA nephropathy model. The chemical structure of ^18^F-ALF-NOTA-PRGD2 is shown in Supplementary Figure S7A. The entire radiosynthesis process took about 30 min with a yield range from 48.99% to 97.33%, which mainly depended on the volume of reaction and the added amount of ^18^F-flouride. The radiochemical purity of the sample could reach 98% (Supplementary Figure S7B). Based on previous reports (Lang et al., 2011; Gao et al., 2012; Chen et al., 2020), the synthesis process of ^18^F-ALF-NOTA-PRGD2 is simple and time-saving.

**FIGURE 4:**
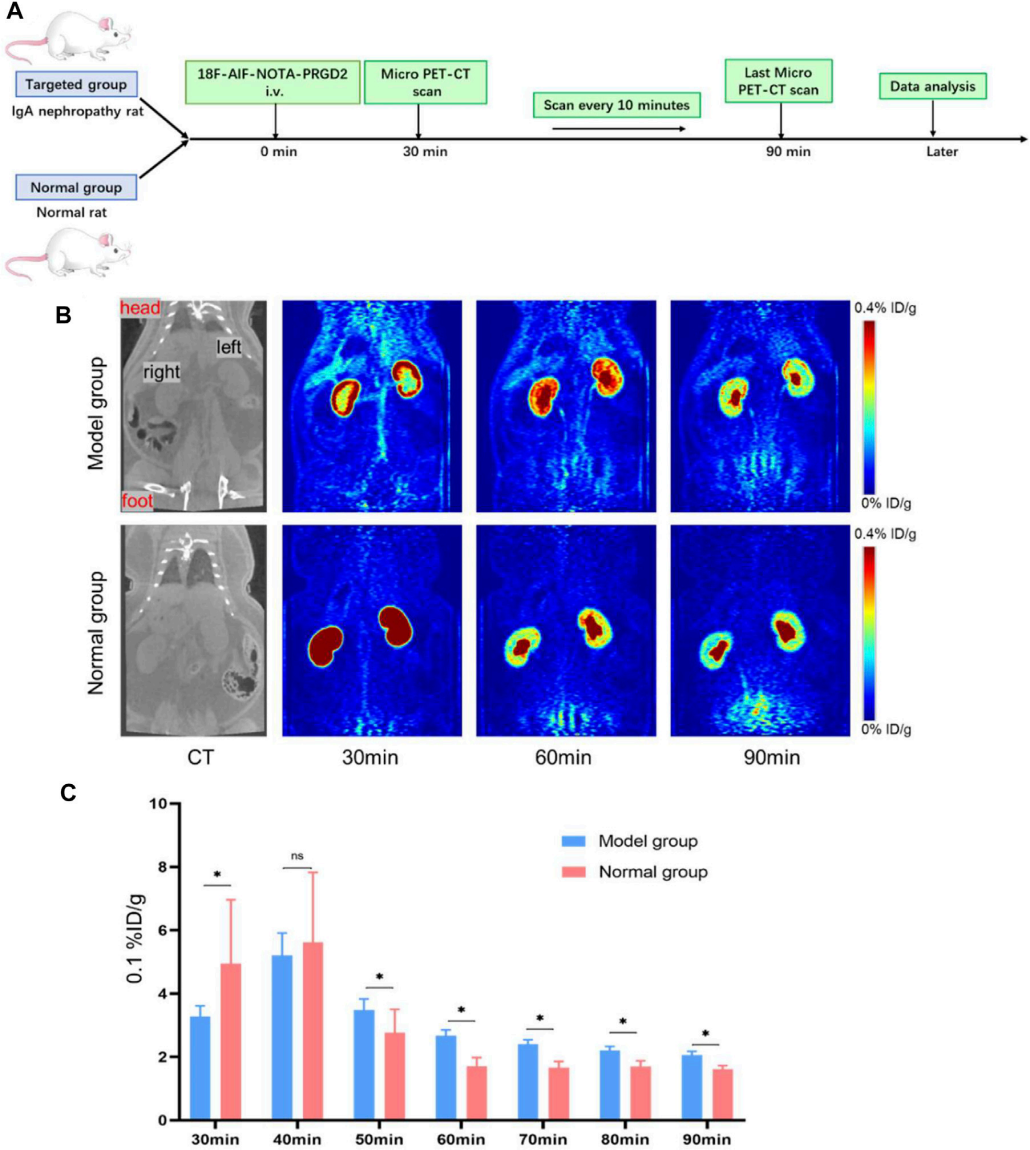
The verification of the IgA nephropathy rat model in micro-PET images with ^18^F-ALF-NOTA-PRGD2. **(A)** Experimental schematic to verify the IgA nephropathy rat model in micro-PET images. **(B)** Micro-PET images at different time points after the injection of ^18^F-ALF-NOTA-PRGD2 for both groups. **(C)** The quantized value of radioactivity at the different time (*p < 0.05).

Subsequently, the^18^F-ALF-NOTA-PRGD2 probe was injected into rats of the IgA nephropathy group and normal group *via* tail vein, and PET imaging was performed (Figures 4A,B). The renal uptakes (%ID/g) of ^18^F-ALF-NOTA-PRGD2 in IgA nephropathy rats were 0.33 ± 0.03, 0.52 ± 0.07, 0.35 ± 0.03, 0.26 ± 0.02, 0.24 ± 0.01, 0.22 ± 0.01, and 0.21 ± 0.01, while those in the normal group were 0.49 ± 0.21, 0.56 ± 0.22, 0.28 ± 0.07, 0.17 ± 0.03, 0.16 ± 0.02, 0.17 ± 0.02, and 0.16 ± 0.01 at 30, 40, 50, 60, 70, 80, and 90 min after injection, respectively. The statistical analysis showed that the mean uptake values in the control group were higher than that in the IgA nephropathy group at 30 and 40 min, while these values were lower in the control group compared with the IgA nephropathy group at 50, 60, 70, 80, and 90 min. There was significant statistical difference of renal radioactive uptake between two groups at 30, 50, 60, 70, 80, and 90 min (*p* < 0.05) (Figure 4C). The former tumor research demonstrated that the uptake of radiotracer depended on the overexpression of integrin αvβ3 (Lee et al., 2013); therefore, IgA nephropathy rats had a higher renal uptake than the control group from 50 to 90 min; the possible reason is that the main factor affecting renal radioactive uptake in the later stage was the expression of integrin αvβ3 after early renal metabolism, which was based on our former study of the overexpression of integrin αvβ3 in IgA nephropathy. This also indicated that the detection of IgA nephropathy through specific molecules targeted to integrin αvβ3 needs a delay phase due to various interferential factors in the early stage. Therefore, the above results further prove the successful establishment of the IgA nephropathy model and provides guidance and recommendations for subsequent MRI.

### MRI Detection of IgA Nephropathy

**FIGURE 5:**
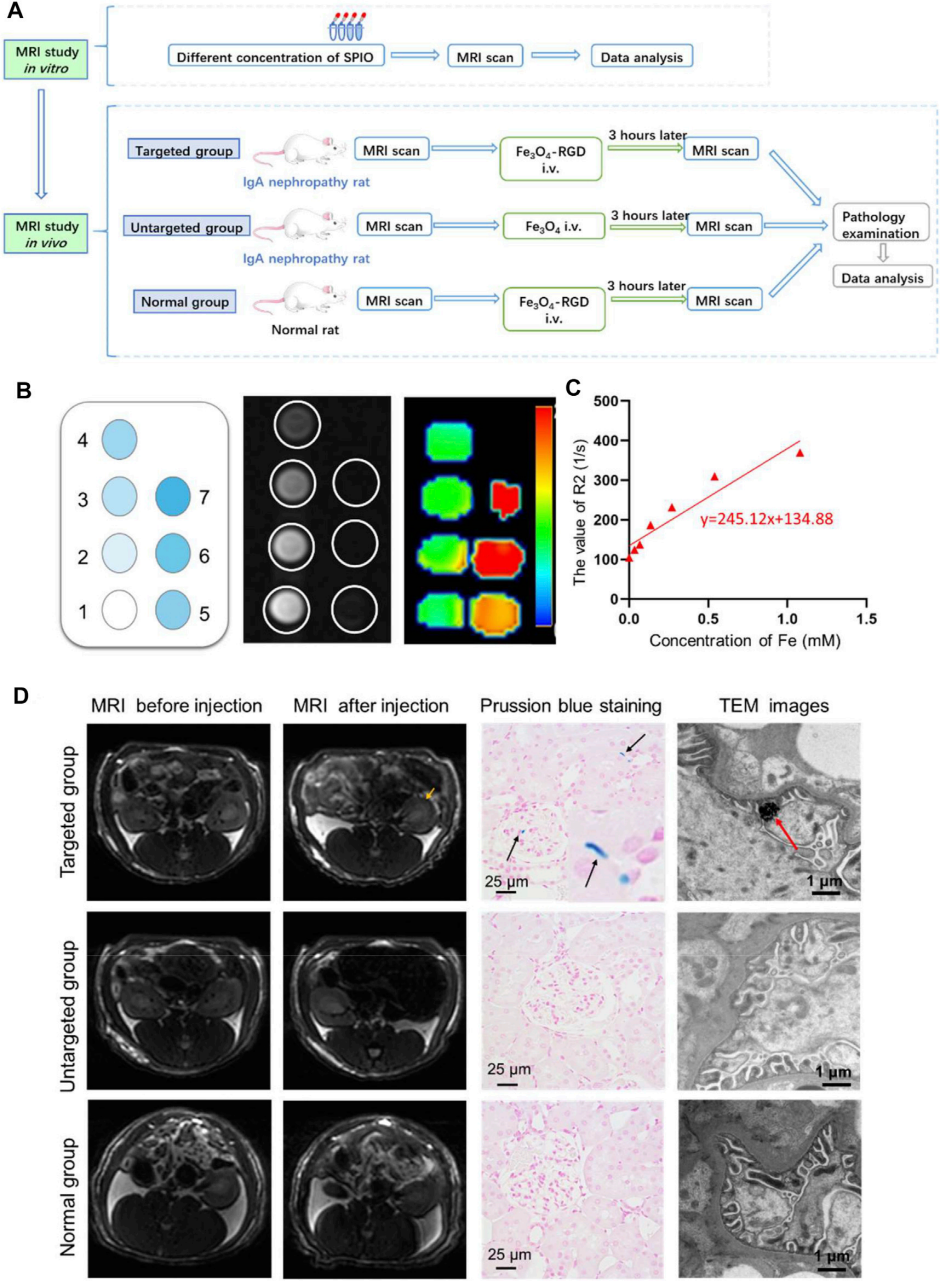
*In vivo* diagnosis of IgA nephropathy by Fe_3_O_4_-RGD NPs. **(A)** Suggested workflow of Fe_3_O_4_-RGD NPs for detecting the IgA nephropathy disease by MRI technology. **(B)** *In vitro* MRI scan of Fe_3_O_4_-RGD with different concentration. **(C)** R2 value variation with the increased concentration of Fe_3_O_4_-RGD. (**D**) The targeting performance of Fe_3_O_4_-RGD for diagnosis of IgA nephropathy. Specifically, T2WI images, kidney Prussian blue staining, and TEM images of rats in each group were carefully investigated.

Next, we studied the MRI of feasibility of Fe_3_O_4_-RGD for IgA nephropathy detection (Figure 5A). First of all, the relaxation efficiency of Fe_3_O_4_-RGD was explored *in vitro*. Fe_3_O_4_-RGD NPs solutions with different Fe concentrations (0, 0.034, 0.067, 0.135, 0.270, 0.540, 1.080) were added into tubes, respectively. Subsequently, the above tubes were performed on the clinical GE 3.0-T MRI for T2-weighted imaging. With the Fe_3_O_4_-RGD concentration increased, the T2WI signal decreased more (Figure 5B), and the R2 value increased as well (Figure 5C). The R2 value of each hole was measured accurately with the READ Y View tool; subsequently, the relevant scatter diagram was drawn and the line was fitted. We could find a linear relationship between the concentration of Fe_3_O_4_-RGD solution and R2 value with r2 value equal to 245.1 mM^−1^ s^−1^. Such remarkable MRI performance indicated the ability of Fe_3_O_4_-RGD as a promising T2WI contrast agent for the exact detection of IgA nephropathy. Furthermore, the circulation time of Fe_3_O_4_-RGD *in vivo* was conducted before performing animal experiments. Our results demonstrated that the blood concentration of Fe_3_O_4_-RGD after intravenous injection decreased slowly over time (Supplementary Figure S8). The ratio of Fe content per gram of blood samples was relatively stable from 0.5 h (0.448 ± 0.028 mg/g) to 6 h (0.399 ± 0.031 mg/g). The ratio for 24 h after injection was 0.366 ± 0.033 mg/g. These results indicated that Fe_3_O_4_-RGD was suitable to apply *in vivo*.

**FIGURE 6:**
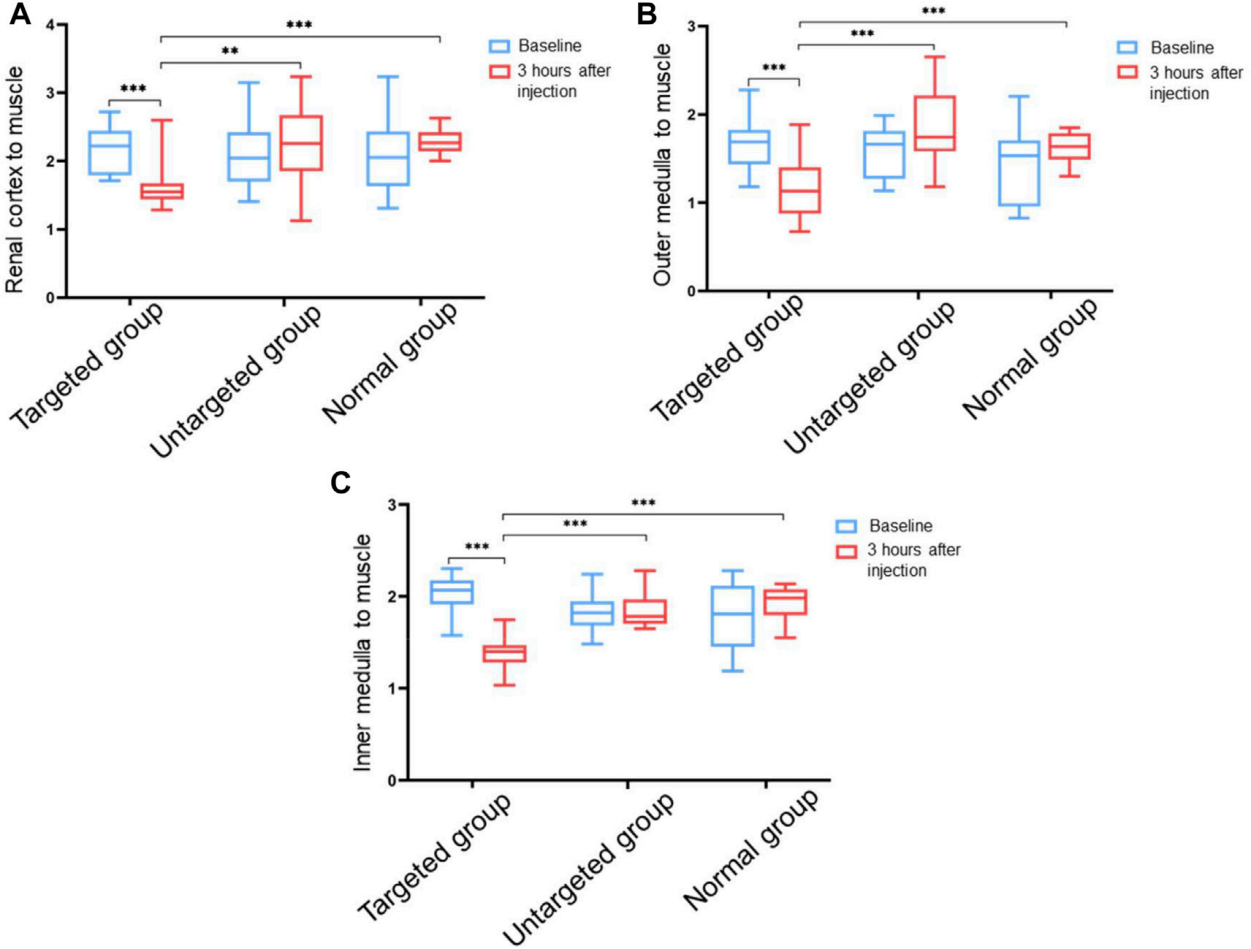
Ratio value of T2WI signals in different tissues of rat kidney to muscle tissue before and after injection of Fe_3_O_4_-RGD for 3 h. **(A)** Ratio value of signal intensity from renal cortex to muscle tissue. **(B)** Ratio value of signal intensity from the outer medulla of the kidney to the muscle tissue. **(C)** Ratio value of signal intensity from the inner medulla of the kidney to the muscle tissue (**p < 0.01, ***p < 0.001).

In order to explore the feasibility of non-invasive diagnosis of IgA nephropathy by MRI through detecting the increased expression of integrin αvβ3 in IgA nephropathy, the experimental rats were divided into three groups (Figure 5A). Group 1 (targeted group) model rats received intravenous tail injection of Fe_3_O_4_-RGD solution with a dose of 15 mg Fe/kg. Group 2 (untargeted group) model rats received intravenous tail injection of Fe_3_O_4_-BSA solution at a dose of 15 mg Fe/kg. Group 3 (normal group) received intravenous tail injection of Fe_3_O_4_-RGD solution at a dose of 15 mg Fe/kg. The results indicated that the T2WI signal of renal parenchyma in the targeted group was lower than baseline after injection of Fe_3_O_4_-RGD for 3 h (Figure 5D). However, compared with baseline signal intensity, no significant decrease of T2WI signal was observed after 3-h injection of Fe_3_O_4_-BSA or Fe_3_O_4_-RGD in the untargeted model group or normal group, respectively (Figure 5D). Furthermore, the quantitative analysis of MRI images was carried out. READ Y View tool was used to measure the T2WI signal intensity of kidney tissues and muscle in each group, and the signal intensity ratio was calculated and analyzed. The signal ratio of renal cortex to muscle, renal outer medulla to muscle, and inner medulla to muscle in the targeted group were significantly lower at 3 h after Fe_3_O_4_-RGD injection than baseline signal intensity (*p* < 0.001) (Figure 6). These results demonstrated that Fe_3_O_4_-RGD NPs were expected to be located in each layer of kidney tissue in the targeted group. Due to the high expression of glomerular integrin αvβ3 in the model rat, confirmed by our experiment (Figure 3B), and anatomical basis of glomerulus and renal tubules mainly distributed in the renal cortex and medulla, respectively (Huang et al., 2015), we can infer that the abundant deposition of Fe_3_O_4_-RGD in the renal cortex is related to the high expression of integrin αvβ3 in glomerulus. Simultaneously, the deposition of Fe_3_O_4_-RGD in the inner and outer medulla may indicate the expression of integrin αvβ3 in renal tubules, which is similar to other integrin molecules such as integrin α3β1 (Pozzi and Zent, 2013). The signal ratios of renal layers to muscle in the targeted group were lower than those in the untargeted group (*p* < 0.05) and in the normal group (*p* < 0.001). All of these indicated that the targeted binding of Fe_3_O_4_-RGD with integrin αvβ3 achieves T2WI signal reduction. One notable fact is that compared with baseline, the mean value of untargeted group and normal group showed a higher signal ratio after the injection of Fe_3_O_4_-RGD NPs; the reason for this is not clear. Because the main metabolism of Fe_3_O_4_-RGD NPs is through liver, spleen, and kidney (Laurent et al., 2008), we infer that the slow muscle metabolism of iron NPs may induce more muscle signal reduction and make the ratio rise after injection; however, this needs to be verified by more experiments.

After the MRI detection process, Prussian blue staining and TEM investigation of renal tissue were further performed to explore the targeted location of iron element in the renal tissue. Prussian blue staining demonstrated that the blue dots were scattered in the glomerulus and tubular interstitium in the targeted group. However, no apparent blue dots were found in the untargeted group and normal group (Figure 5D). The renal expression of integrin αvβ3 in the IgA model group was higher than the normal group. The Fe_3_O_4_-RGD NPs can be well combined with renal integrin αvβ3 in the targeted group. Thus, no iron element was observed in the untargeted group and normal group for Prussian blue staining. Moreover, the deposition of black iron oxide NPs was observed in the foot process of the targeted group by TEM, while no black iron oxide NPs were detected in the untargeted group and normal group. Finally, we have used the ICP to quantify iron content. This result exhibited that the ratio of Fe content to kidney was 0.240 ± 0.002 mg/g for the administration group after injection of Fe_3_O_4_-RGD for 3 h, and the ratio was 0.130 ± 0.010 mg/g for the normal group. These results indicated that the deposition of Fe_3_O_4_-RGD in kidney for the administration group was higher than that for the normal group (Supplementary Figure S9). Besides, the ratio difference was statistically significant (*p* < 0.05). Therefore, the above data further illustrated the dominant location of Fe_3_O_4_-RGD in the glomerular podocytes’ reduced kidney T2WI signal of the rats with IgA nephropathy.

## Conclusion

In this study, we have developed a sensitive, specific, and biocompatible integrin αvβ3-targeted superparamagnetic Fe_3_O_4_ NPs for the noninvasive MR imaging of integrin αvβ3 in IgA nephropathy disease. The rat model was successfully established and verified by biochemical tests and histological staining. Furthermore, the micro-PET ^18^F-AlF-NOTA-PRGD2 probe molecule was utilized to confirm the successful establishment of the IgA nephropathy in the rat model. Subsequently, the synthesized RGD-Fe_3_O_4_ NPs were injected intravenously into model rats and the integrin αvβ3-targeted T2WI RGD-Fe_3_O_4_ NPs proved the accurate detection of the IgA nephropathy disease. Therefore, our study demonstrated the clinical possibility of utilizing the safe and effective Fe_3_O_4_-RGD for accurate MR imaging to diagnose IgA nephropathy noninvasively.

## Data Availability

The raw data supporting the conclusions of this article will be made available by the authors, without undue reservation.
