# Supplementary material for: The Feasibility of Targeted Magnetic Iron Oxide Nanoagent for Noninvasive IgA Nephropathy Diagnosis
Source: Front Bioeng Biotechnol. 2021 Nov 25;9:755692. doi: 10.3389/fbioe.2021.755692 (PMC8656216; doi:10.3389/fbioe.2021.755692)
Supplement: Supplementary file 1 [file DataSheet1.pdf]

Supplementary Material

Materials

Bovine Serum Albumin (A8010) and Lipopolysaccharides (L8880) was purchased from Solarbio, Beijing, China. Castor oil (C805202) was generated from MACKLIN, Shanghai, China. Anti-Integrin alpha V beta 3 antibody (ab7166) was purchased from Abcam, Cambridge, Britain. Cyclo-RGDfK (A8164) was purchased from APExBIO Technology LLC, Houston, America. BSA coating Fe3O4 nanoparticles (10nm) (R-CZ10010) was acquired commercially from Xi'an ruixi Biological Technology Co., Ltd, Shanxi, China. 2.5% glutaraldehyde (R20510) was purchased from Yuanye Biotechnology Co., Ltd, Shanghai, China. 10% neutral buffer formalin was purchased from Tonghe Biotechnology Co., Ltd, Jinghua, Zhejiang, China. Carbon tetrachloride (CCl4) and 37% hydrochloric acid was provided by the Animal Laboratory Center of the first affiliated hospital of Zhejiang University Medical College. Other reagents were purchased from Sinopharm Chemical Reagent Co., Ltd. Shanghai, China.

TEM investigation:

To investigate the immune complex deposition in rat kidney, the renal sections were fixed in 2.5% glutaraldehyde overnight at 4℃, and fixed in 1% Russian acid solution for 1 hour. After that, samples washed with PBS for three times. Renal tissues were dehydrated with gradient ethanol solutions (including 30%, 50%, 70%, 80%, 90%, 95% and 100%) for 15-20min, and treated with pure acetone for 20 min. The mixture of Spurr embedding agent and acetone (1:1), the mixture of Spurr embedding agent and acetone (3:1), as well as pure Spurr embedding agent were treated samples for 1 h, 3 h and overnight respectively. After heating at 70 °C overnight, the samples were sliced and obtained 70-90 nm slices. The slices were stained with lead citrate solution and 50% ethanol saturated solution of uranyl acetate for 5-10 min. Finally, the samples were observed by TEM (H-7650, Hitachi, Tokyo, Japan).

Synthesis of ^{18}F-AlF-NOTA-PRGD2

Synthesis of ^{18}F-AlF-NOTA-PRGD2 was based on the previous literature [Lang et al., 2011] with some adjustments. 4 mg NOTA-PRGD2 was accurately weighed and dissolved in 640uL of pure water in a 2mL centrifuge tube and mixed well. Then, 40 uL of NOTA-PRGD2 solution, 6uL of 0.01mM AlCl3 solution, 5 μL of glacial acetic acid, as well as 324 uL of DMF was added into a 2 mL centrifuge tube and mixed sufficiently. 0.5mL ^{18}F fluoride (about 30mCi) was added into the mixture, and then heat it at 100 °C for 10 min. After cooling, for purifying the mixture, it was transferred to C18 column with 4 mL water, washed with 20 mL water for 3 times, and finally eluted with 1 mL ethanol to acquire product. A little of product was taken for HPLC analysis to detect the radiochemical yield and purity.

Stability of ^{18}F-AlF-NOTA-PRGD2

The stability of ^{18}F-ALF-NOTA-PRGD2 was performed by paper chromatography. Firstly, 1 mL of fetal bovine serum and 1mL of PBS buffer were added into two 2 mL centrifuge tubes respectively.

Then, 400 μL product was added and mixed at room temperature. At 0, 1, 2, 4, 6 h, a little of mixture were pointed on two pieces of filter papers respectively, and hanged in chromatography cylinder with acetone solution. Finished that, the filter papers were taken out, dried and divided into 10 parts. Finally, radioactivity was measured by gamma counter.

Cytotoxicity of the Fe_3O_4-RGD

The cytotoxicity of Fe_3O_4-RGD was assessed by Cell Counting Kit 8 (CCK-8) (APExBIO, Houston, TX, USA) assay. 293T cells (ATCC, Rockville, MD, USA) as a kind of normal cells were cultured in DMEM containing 10% fetal bovine serum (FBS) in a 37 °C incubator with 5% CO2. And then, 293T cells were seeded into 96-well plates (5×10^3/well), and cultured at 37 °C with 5% CO_2 atmosphere for 24 h. After that, a series concentration (0, 10, 20, 40 and 80 μg/mL) of Fe_3O_4-RGD were added into wells and incubated for 24 h. Then, 90 μl of medium and 10 μl of CCK-8 reagent were added and incubated for another 1 h. The wells contain cells, medium, CCK-8 reagent and Fe3O4-RGD solution were set as sample group. Control group contained cells, medium and CCK-8 reagent. The wells contained only medium and CCK-8 reagent were labeled as blank group. Absorbances values (OD values) of each well were measured at 490 nm via a microplate absorbance reader (Epoch 2, BioTek, Winooski, VT, USA). The viability was calculated as follow:

Cell viability (%) = $(OD_{sample}-OD_{blank}/OD_{control}-OD_{blank}) \times 100\%$.

Hemolysis assay

To evaluate the hemocompatibility of the Fe_3O_4-RGD through hemolysis assays, 2mL of SD rat blood was collected and centrifuged at 1500 rpm for 15 minutes. The plasma was removed. The erythrocytes were washed and diluted with PBS. 800 μL of Fe_3O_4-RGD solutions (6.25, 12.5, 25, 50, 100, 200 μg/mL) as samples were added to 200 μL of diluted erythrocytes. For positive control, 800 μL of distilled water was added into erythrocytes solution. And, 800 μL of PBS was added into erythrocytes solution as negative control. These tubes were incubated at 37 °C for 1 h. Each tube was centrifuged at 12000 rpm for 15 minutes. The supernatant was collected for measuring the absorbance at 540 nm with a microplate reader (Epoch 2, BioTek, Winooski, VT, USA). The hemolysis ratio was calculated with following equation:

Hemolysis ratio (%) = $[(OD_{samples}-OD_{negative\ control})/ (OD_{positive\ control}-OD_{negative\ control})] \times 100\%$

Blood circulation of Fe_3O_4-RGD

3 Male SD rats were bought from Zhejiang Academy of Medical Sciences and fed with 22.8°C room temperature and 59.6% relative humidity level in the SPF degree animal laboratory of the First Affiliated Hospital, College of Medicine, Zhejiang University. The model SD rats (n=3) were anesthetized with 4% chloral hydrate at a dose of 0.8 ml/kg by intraperitoneal injection and then were administrated with Fe_3O_4-RGD at a dose of 15 mg/kg *via* intravenously injection. After that, blood

sample from rat eyes were collected at preset time points (0, 0.5, 1, 2, 4, 6, 12, 24 h). ICP-MS (Agilent 7700, Agilent Technologies, California, USA) was used for measuring the Fe content of blood samples. And the ratio of Fe content to per gram blood samples were calculated.

Renal biodistribution of Fe_3O_4-RGD

The model SD rat (n=1) was anesthetized with 4% chloral hydrate at a dose of 0.8 ml/kg by intraperitoneal injection and then was injected with Fe_3O_4-RGD (15mg/kg) as administration group. After injection for 3 h, the SD rat was sacrificed through cervical dislocation and the kidney were collected. Besides, a health SD rat was not accepted any management and took cervical dislocation to collect kidneys as normal group. The Fe content deposition of these kidneys were detected by ICP-MS (Agilent 7700, Agilent Technologies, California, USA). The ratio of Fe content to per gram kidney was calculated.

Statistical Analysis

Statistical analysis was performed by using SPSS 26 (IBM Corp, Armonk, NY). T test was applied for the comparison in intragroup when the data followed normal distribution, otherwise, Mann-Whitney U test was performed. One Way ANOVA test was used to evaluate the intergroups correlations. $P<0.05$ were considered significant.

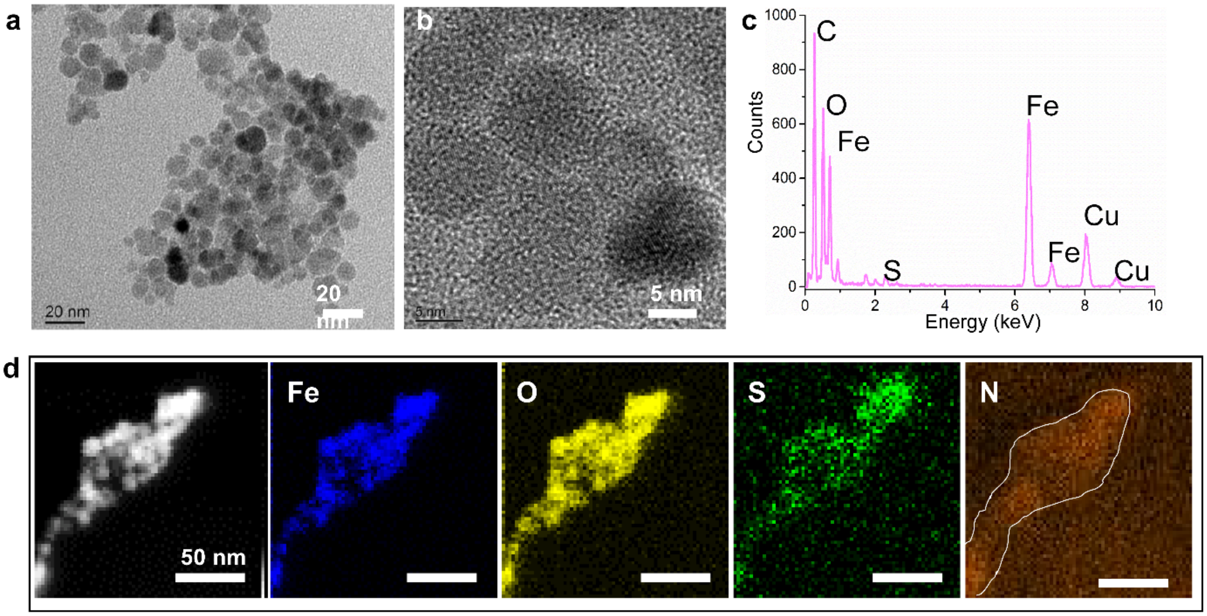

Figure S1 Characterization of the RGD molecules modified magnetic nanoparticles (Fe_3O_4-RGD NPs) (**a, b**) TEM and magnified TEM images of Fe_3O_4-RGD. (**c, d**) HAADF-STEM elemental mapping and EDX patterns images of Fe_3O_4-RGD.

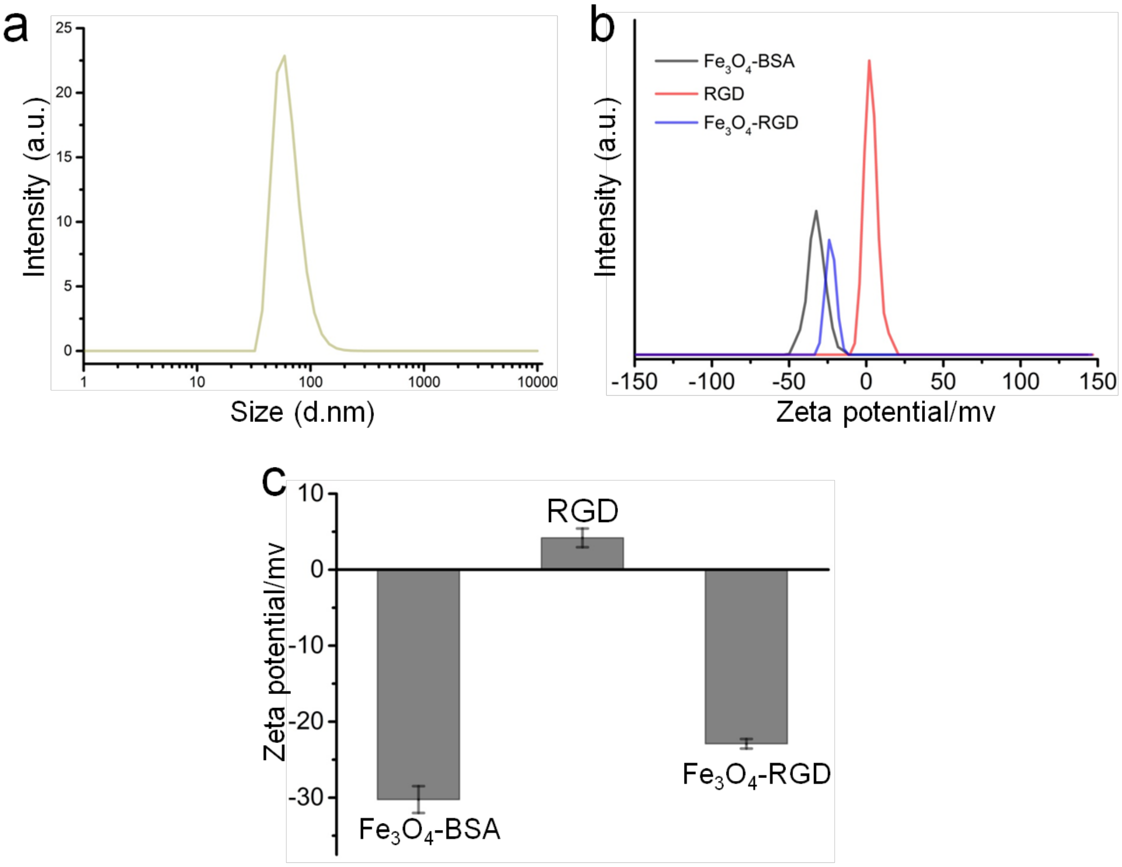

Figure S2 (**a**) Size distribution of Fe_3O_4-RGD nanoparticles in ultrapure aqueous solution. (**b,c**) Zeta potential of Fe_3O_4-RGD nanoparticles during the RGD molecule modification process

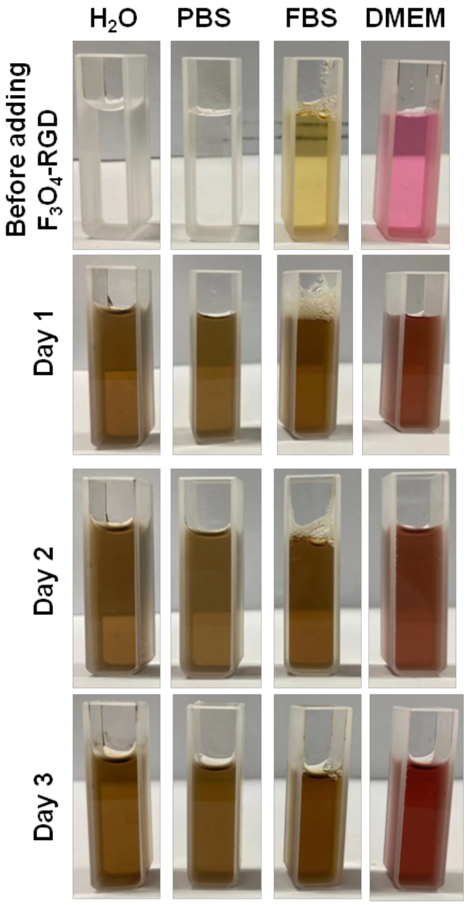

Figure S3 The photograph stable performance of Fe_3O_4-RGD nanoparticles in different media solution for different time points.

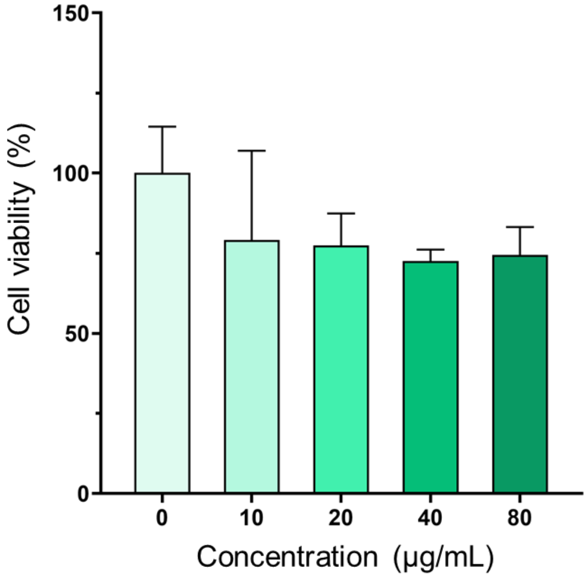

Figure S4 Cytotoxicity of a series concentration of Fe_3O_4-RGD (0, 10, 20, 40, 80 μg/mL) for 293T cells (normal cell) by CCK-8 assay *in vitro* with 24h incubation.

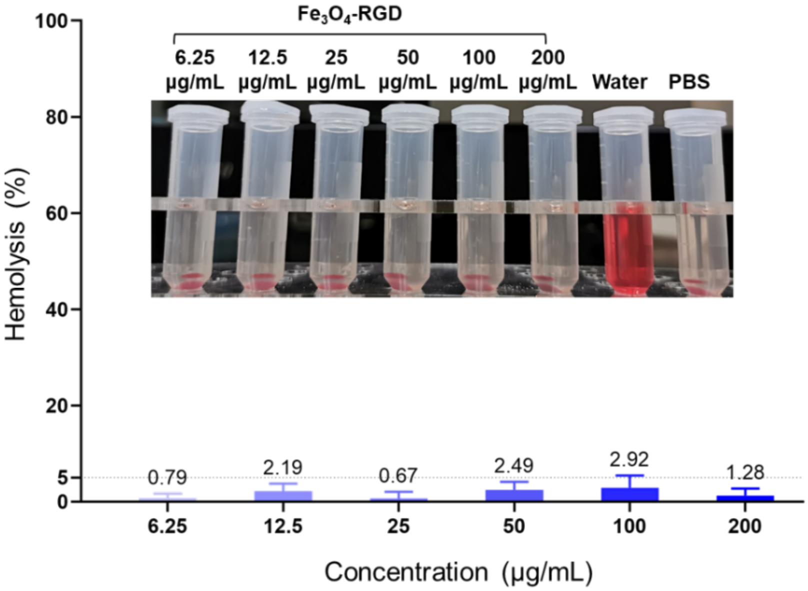

Figure S5 The hemolysis assay of Fe_3O_4-RGD samples to SD rat blood. The above figure showed that positive control group presented red liquid comparing with faint-yellow liquid of Fe_3O_4-RGD samples (6.25, 12.5, 25, 50, 100, 200μg/mL) and negative control group. All the hemolysis rate for Fe_3O_4-RGD samples were lower than 5%.

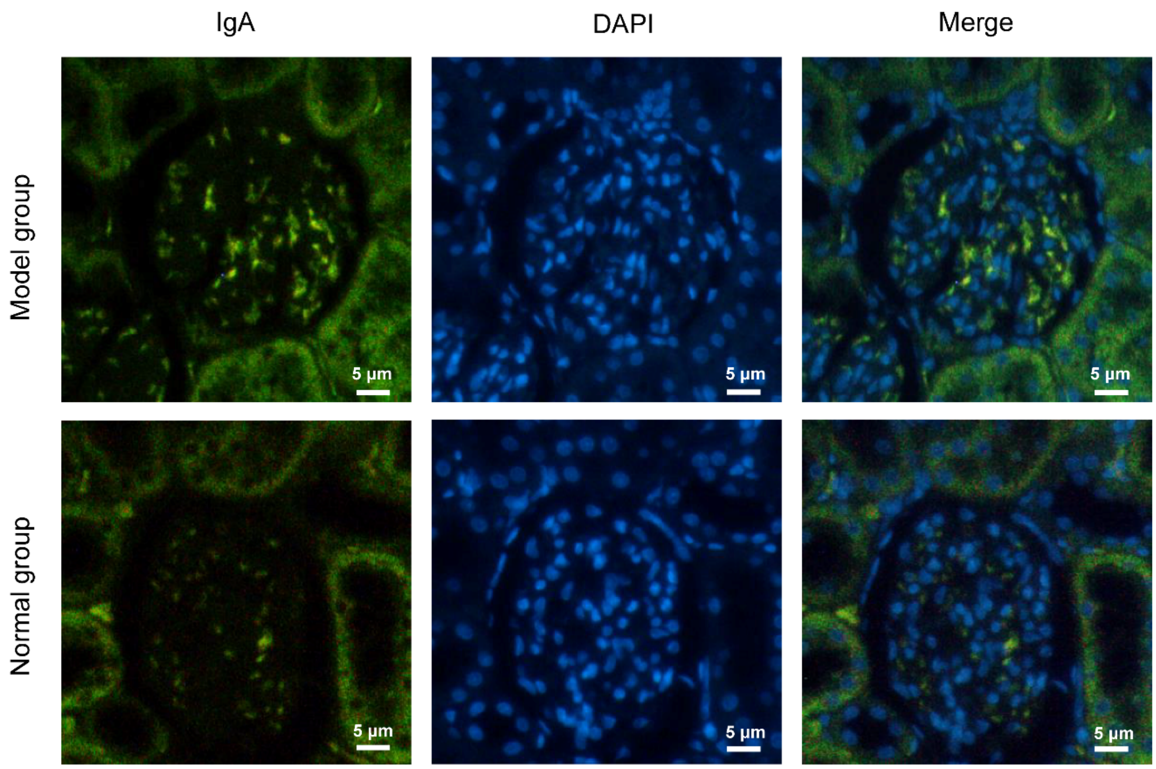

Figure S6 IgA Immunofluorescence of renal tissue with different treatment. There were a lot of IgA molecule expression in glomerular mesangial area for model group. A small amount of IgA molecule was found in the glomerular mesangial area of the normal group.

a

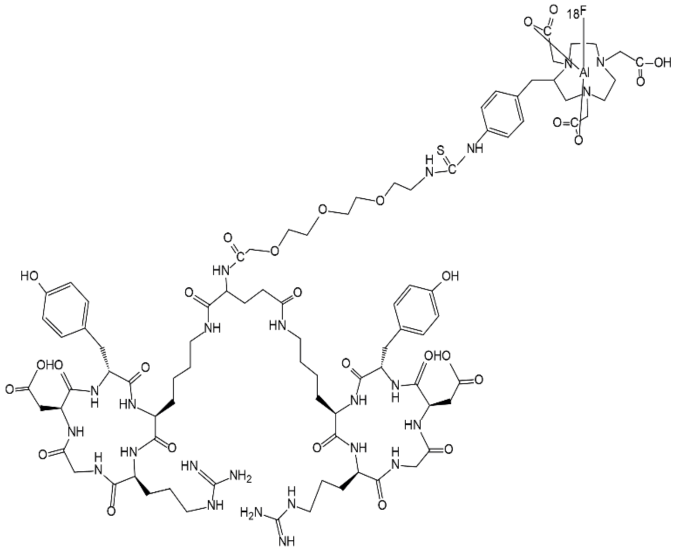

b

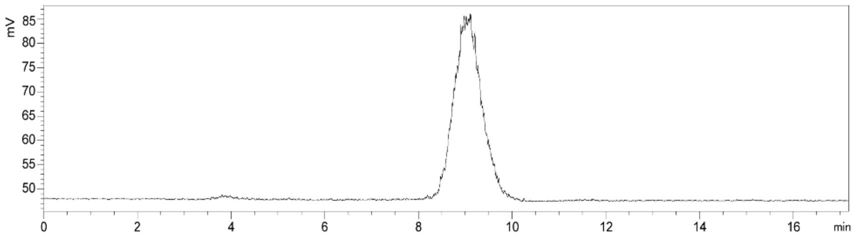

Figure S7. The chemistry and radiochemistry of ^{18}F-ALF-NOTA-PRGD2. (**a**) Chemical formula and structural of ^{18}F-ALF-NOTA-PRGD2. (**b**) The retention time calculation of ^{18}F-ALF-NOTA-PRGD2 was 9.1 min, and the radiochemical purity was about 98.8%.

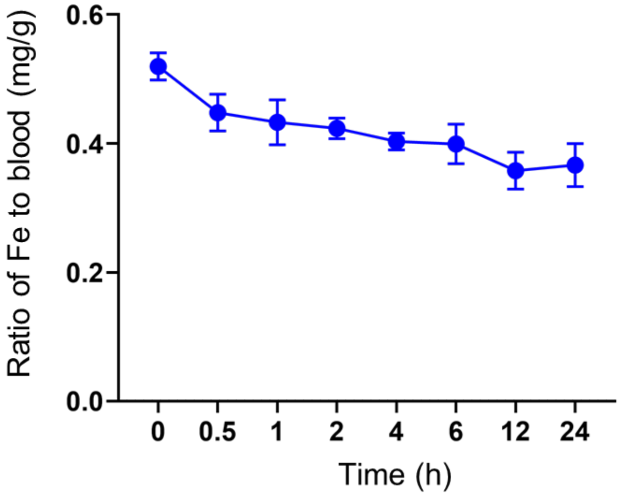

Figure S8 The 24 h pharmacokinetic curves of Fe_3O_4-RGD in SD rats through intravenous injection.

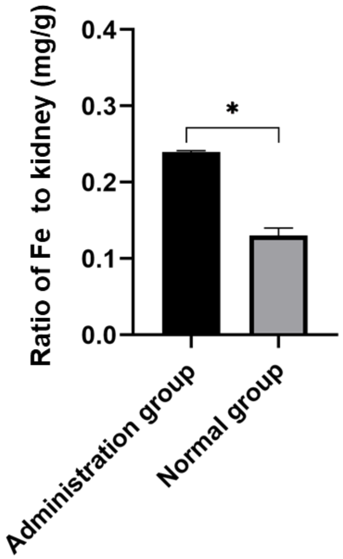

Figure S9 The ratio of Fe content to per gram kidney was statistical significance ($p<0.05$) between administration to normal group.

References

Lang, L., Li, W., Guo, N., Ma, Y., Zhu, L., Kiesewetter, D.O., et al. (2011). Comparison study of [18F]FAl-NOTA-PRGD2, [18F]FPPRGD2, and [68Ga]Ga-NOTA-PRGD2 for PET imaging of U87MG tumors in mice. *Bioconjug Chem* 22(12), 2415-2422. doi: 10.1021/bc200197h.
